# High-level expression of a novel liver-targeting fusion interferon with preferred *Escherichia coli* codon preference and its anti-hepatitis B virus activity in *vivo*

**DOI:** 10.1186/s12896-015-0177-1

**Published:** 2015-06-12

**Authors:** Xuemei Lu, Jie Wang, Xiaobao Jin, Jiayong Zhu

**Affiliations:** School of Basic Courses, Guangdong Pharmaceutical University, 280 Wai Huan Dong Road, Guangzhou Higher Education Mega Center, Guangzhou, People’s Republic of China; Guangdong Provincial Key Laboratory of Pharmaceutical Bioactive Substances, 280 Wai Huan Dong Road, Guangzhou Higher Education Mega Center, Guangzhou, People’s Republic of China

**Keywords:** Preferred codon usage, Induction conditions optimize, Recombinant liver-targeting fusion interferon, HBV transgenic mice

## Abstract

**Background:**

In our previous study, a novel liver-targeting fusion interferon (IFN-CSP) combining IFN α2b with *plasmodium* region I peptide was successfully constructed. IFN-CSP has significant inhibition effects on HBV-DNA replication in HepG2.2.15 cells. The aim of the present investigation was focused on how to produce high levels of recombinant IFN-CSP and its *in vivo* anti-hepatitis B virus (HBV) activity.

**Methods:**

A modified DNA fragment encoding IFN-CSP was synthesized according to *Escherichia coli* (*E. coli*) preferred codon usage and transformed into *E. coli* BL21 (DE3) for protein expression. The induction conditions were systematically examined by combining one-factor experiments with an orthogonal test (L(9)(3)(4)). The antigenicity of the purified protein was characterized by western blot analysis. The *in vivo* tissue distribution were assayed and compared with native IFN α2b. HBV-transgenic mice were used as *in vivo* model to evaluate the anti-HBV effect of the recombinant IFN-CSP.

**Results:**

The results showed that the *E. coli* expression system was very efficient to produce target protein.

**Conclusion:**

Our current research demonstrates for the first time that IFN-CSP gene can be expressed at high levels in *E. coli* through codon and expression conditions optimization. The purified recombinant IFN-CSP showed liver-targeting potentiality and anti-HBV activity *in vivo*. The present study further supported the application of IFN-CSP in liver-targeting anti-HBV medicines.

## Background

Over the past decade, a number of functional proteins have been produced successfully by recombinant DNA methods [1, 2]. The most commonly used host cell is *Escherichia coli* (*E. coli*) because of high yield and low cost [3]. However, high-level expression of recombinant proteins using *E. coli* as the host cell has been affected by some factors, such as the different preferential codons in different expression systems and various induction conditions of target protein [4, 5].

The interferons (IFNs) are a family of protein with the ability to induce antiproliferative, immunomodulatory, and antiviral activities [6–8]. For more than a decade, interferon therapy is the gold standard in treatment for certain forms of viral hepatitis and carcinogenesis [9]. However, therapeutic efficacy has been limited because interferon does not have organ-specific affinity and its *in vivo* half-life is short [10]. Incorporation of *plasmodium* region I peptide was demonstrated to be a promising strategy for the development of liver-targeting drug [11, 12]. In our previous study, a novel liver-targeting fusion interferon (IFN-CSP) combining IFN α2b with *plasmodium* region I peptide was successfully designed [13]. The results of *in vitro* anti-hepatitis B virus (HBV) activity of IFN-CSP showed that IFN-CSP has significant inhibition effects on HBV-DNA replication in HepG2.2.15 cells [14]. *In vitro* liver tissue binding analysis revealed that IFN-CSP specific targeting to liver tissue [13]. IFN-CSP may be an excellent candidate as a liver-targeting anti-HBV agent. However, the *in vivo* tissue distribution and anti-HBV activity of IFN-CSP requires further investigate. Moreover, it is desirable to produce IFN-CSP in a large scale for therapeutic application.

The main purpose of the present investigation was focused on how to produce high levels of recombinant IFN-CSP in a cost-effective way. We also investigated the *in vivo* tissue distribution and anti-HBV activity of IFN-CSP. Therefore, a modified DNA fragment encoding the IFN-CSP was synthesized by overlapping extension-PCR method according to *E. coli* preferred codon usage. The IFN-CSP gene was cloned into the bacterial expression vector pET-21b and transferred into the expression strain *E. coli* BL21. The suitable induction conditions were systematically optimized by combining one-factor experiments with an orthogonal experiment (L(9)(3)(4)). The antigenicity of the purified protein was characterized by western blot analysis. The *in vivo* tissue distribution were assayed and compared with native IFN α2b. HBV-transgenic mice were used as *in vivo* model to evaluate the anti-HBV effect of the recombinant IFN-CSP.

## Methods

### Pasmids, strains and culture media

pMD20-T (Takara, Japan) was used for gene cloning. pET-21b (Novagen, USA) was employed to construct expression vector. *E. coli* strain DH5α (Novagen, USA) was applied as the host for gene manipulation. *E. coli* strain BL21 (DE3; Novagen, USA) served as expression host for fusion protein. Luria-Bertani (LB) medium was used for bacterial growth and protein expression.

### Construction of the liver-targeting fusion interferon gene using *E. coli* preferred codon

To improve the expression level of IFN-CSP in *E. coli*, a new IFN-CSP coding sequence was designed. The optimized codons usage pattern of *E. coli* genes was employed according to the codon usage pattern of *E. coli* (http://www.kazusa.or.jp/codon/). Based on the method of polymerase chain reaction (PCR)-based gene synthesis and gene splicing by overlap extension, a modified three-step method [15], called “splicing by overlapping extension-PCR (SOE-PCR)”, was adopted to construct the synthetic IFN-CSP gene. We designed 16 oligonucleotides encoding for the IFN-CSP protein. The sense and antisense oligonucleotides with 19 bp complementary overlapping sequence are presented in Table 1.

**Table 1:** Nucleotide sequences of oligonucleotides designed for assembly of IFN-CSP^*^

Primers	Location	Nucleotide sequence (from 5′ end to 3′ end)
SR-1	1–29	GGAATTCCATATG *TGTGATCTGCCTCAAA*
AR-1	14–71	*GTGCCAGGAGCATCAAGGT*ACGACGGCTACCCAGGCTGT*GGGTTTGAGGCAGATCACA*
SR-2	53–110	*ACCTTGATGCTCCTGGCAC*AGATGCGTCGTATCTCTCTT*TTCTCCTGCTTGAAGGACC*
AR-2	92–149	*GGTTGCCAAACTCCTCCTG*TGGAAATCCAAAGTCATGAC*GGTCCTTCAAGCAGGAGAA*
SR-3	131–188	*CAGGAGGAGTTTGGCAACC*AGTTCCAAAAGGCTGAAACC*ATCCCTGTCCTCCATGAGA*
AR-3	170–227	*AGTCCTTTGTGCTGAAGAG*ATTGAAGATCTGCTGGATCA*TCTCATGGAGGACAGGGAT*
SR-4	209–266	*CTCTTCAGCACAAAGGACT*CATCTGCTGCTTGGGATGAG*ACCCTCCTTGACAAATTCT*
AR-4	248–305	*AGGCTTCCAGGTCATTCAG*CTGCTGGTAGAGTTCAGTGT*AGAATTTGTCAAGGAGGGT*
SR-5	287–344	*CTGAATGACCTGGAAGCCT*GTGTGATTCAGGGGGTGGGG*GTGACAGAGACTCCACTGA*
AR-5	326–383	*GGAAGTATTTACGCACAGC*CAGAATGGAGTCCTCCTTCA*TCAGTGGAGTCTCTGTCAC*
SR-6	365–422	*GCTGTGCGTAAATACTTCC*AACGTATCACTCTCTATCTG*AAAGAGAAGAAATACAGCC*
AR-6	404–461	*AACGCATGATTTCTGCACG*GACAACCTCCCAGGCACAAG*GGCTGTATTTCTTCTCTTT*
SR-7	443–500	*CGTGCAGAAATCATGCGTT*CTTTTTCTTTGTCAACAAAC*TTGCAAGAAAGTTTACGTA*
AR-7	482–525	*CGTAATTTCTCGTTGTCTT*CCTTAC*TACGTAAACTTTCTTGCAA*
SR-8	507–561	*AAGACAACGAGAAATTACG*TAAACCAAAACATAAAAAA*TTAAAGCAACCAGCGGA*
AR-8	545–578	CCGCTCGAG **ATTA**ACCA*TCCGCTGGTTGCTTTAA*

^*^Letters in italic denote the overlapped parts in IFN-CSP. Restriction sites are underlined. Bold type indicates the additional terminal codon ‘TAAT’

### Construction of expression plasmids IFN-CSP/pET-21b

**Fig. 1:**
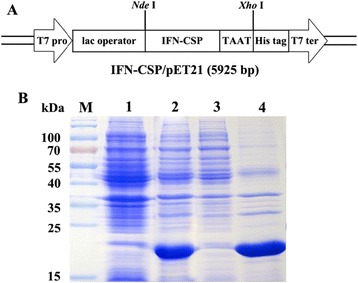
Schematic diagram of *IFN-CSP* gene in the expression vector IFN-CSP/pET-21b and expression of IFN-CSP protein in *E. coli* BL21/pET-21b-IFN-CSP. **a**: A Schematic diagram of IFN-CSP/pET-21b (T7 pro, T7 promoter; T7 ter, T7 terminator). **b**: SDS-PAGE analysis of protein expression. Lane M: Protein molecular weight marker, Lane 1–2: Total proteins of *E. coli* BL21/pET-21b-IFN-CSP before and after induction, Lane 3–4: Supernatant and precipitation after ultrasonication and centrifugation

The synthetic IFN-CSP gene fragments were cloned into the pMD20-T vector (Takara) and transformed into *E. coli* DH5a according to the procedures described by the manufacturer. The generated recombinant plasmids IFN-CSP/pMD20-T were digested and the inserts were cloned into *Nde* I/*Xho* I restriction sites of the expression vector pET-21b (Fig. 1a). The resulting expression plasmid IFN-CSP/pET-21b was finally transformed into *E. coli* BL21 (DE3) for IFN-CSP expression.

### Optimization of IFN-CSP expression

To improve the expression level of IFN-CSP, the induction conditions [16] like cultivation temperature, induction timing, inducer concentrations, induction time were systematically examined by combining one-factor experiments with an orthogonal test (L(9)(3)(4)). A fresh clone of recombinant *E. coli* BL21 with plasmid IFN-CSP/pET-21b grew in Luria-Bertani (LB) medium containing 100 μg/ml ampicillin. Different conditions of induction (temperatures: 17 °C, 22 °C, 27 °C, 32 °C, 37 °C, 42 °C; OD_600_: 0.1, 0.2, 0.4, 0.6, 0.8, 1.0, 2.0; IPTG concentrations: 0.1, 0.2, 0.4, 0.6, 0.8, 1.0, 1.2, 1.5 mM; induction times: 1, 2, 4, 6, 8, 10, 12, 24 h) were conducted to optimize the expression of heterologous protein in *E. coli*. To evaluate the expression level of IFN-CSP, the cells were harvested by centrifugation at 10,000 rpm for 10 min. Samples were analyzed by 15 % sodium dodecyl sulfate polyacrylamide gel electrophoresis (SDS-PAGE) and the percentage fraction of proteins was assessed by densitometric using Gel-Pro analyzer Version 4.5 software. Total protein concentration was determined by Bradford method and the concentration of IFN-CSP was calculated according the percentage fraction and total protein concentration.

### Purification, antigenicity and biological activity analysis

Cells were collected by centrifugation. After lysis by ultrasonication in an ice bath, an improved seven-step process was conducted to obtain purified IFN-CSP based on previously described methods [13, 15, 17]. Briefly, cellular pellet was separated from the cell lysate by centrifugation at 6000 g and 4 °C for 10 min. IB material was washed with 1 % Triton X-100 and 2 M urea (containing 2 % deoxycholate), and then dissolved in 6 M guanidine hydrochloride (GuHCl) (containing 2.5 mM DTT and 50 mM Tris-HCL buffer, pH 8.0). The solubilized IFN-CSP was refolded by dialysis with the 50 mM Tris-HCl buffer systems (containing 0.2 mM glutathione oxidized and 2 mM glutathione reduced), followed by changes of the buffer containing decreasing GuHCl concentrations (4, 2 and 1 M) and finally with Tris-HCl buffer (pH 7.4). The refolded IFN-CSP was purified by heparin affinity chromatography according to the instruction of HiTrap affinity column (GE healthcare, USA). The IFN-CSP was eluted with a linear gradient of 0.1–2 M NaCl. After dialysis against the Tris-HCl buffer, the sample was further purified to remove endotoxin by polymyxin B column (Bio-Rad, USA). After detecting the lipopolysaccharides (LPS) content with the chromogenic limulus amoebocyte lysate assay (Associates of Cape Cod, USA), the purified IFN-CSP was lyophilized and stored at -80 °C.

The antigenicity of the purified protein was characterized by western blot analysis. We performed western blot with primary goat polyclonal anti-IFN α antibody (1:200; Santa Cruz Biotechnology, USA) and peroxidase-conjugated rabbit anti-goat IgG (1:2500; Santa Cruz Biotechnology, USA) as previously described [13]. The purity of the purified protein was assessed by reverse phase high-performance liquid chromatography (RP-HPLC) on a C18 column (250 × 4.6 mm, 5 μm, and 300 Å, Agilent, USA) in an analytical Alliance HPLC System (Waters, USA). The molecular weight of purified IFN-CSP was characterized by mass spectrometry on a matrix-assisted laser desorption/ionization (MALDI) mass spectrometer (Applied Biosystems, USA).

Biological activity of IFN-CSP was determined in a standard cytopathic effect inhibition assay using vesicular stomatitis virus (VSV)/human amniotic cells (WISH) measure system according to China Biologicals Requirements.

### *In vivo* tissue distribution experiment

All animal experimental protocols were approved by the Guangdong Pharmaceutical University Animal Care and Use Committee. Normal Balb/c mice (18–22 g) were purchased from the Center for Experimental Animals of Guangdong Province (Guangzhou, China). Balb/c mice (n = 90) were injected via the tail vein with 9.01 μg/kg (0.46 nmol/kg) of control IFN α2b or 10.09 μg/kg (0.46 nmol/kg) of recombinant IFN-CSP. At 30, 60, 120, 240 and 480 min post injection, the mice were anesthetized, euthanized and then the liver, heart, spleen, lung and kidney were excised, rinsed with saline, dried and weighed. The concentration of IFN α2b was determined by ELISA (Shanghai Senxiong Biotechnology Limited Corporation.) according to the manufacturer.

### *In vivo* anti-HBV experiment

Balb/c-HBV transgenic mice (18–22 g) were purchased from Infectious Disease Center of No. 458 Hospital (Guangzhou, China) and kept under the pathogen free condition in the facility of the Guangdong Pharmaceutical University. The Balb/c-HBV transgenic mice were randomly assigned to either treatment or control groups (n = 6). Six non-transgenic Balb/c mice were used as normal control mice. Each mouse was intramuscular injected with IFN-CSP (at dose of 10^1^ U/g body weight, 10^2^ U/g body weight, 10^3^ U/g body weight, respectively) once a day for 28 days. Sterile physiological saline was used as control. IFN α2b (10^3^ U/g body weight) was used as drug control. Mice were sacrificed at day 28. Serum samples and livers were collected.

### Measurement of serum HBsAg and HBV-DNA

Serum HBsAg was measured by enzyme-linked immunosorbent assay (ELISA) using a HBV diagnostic kit (Shanghai Kehua Biotech Co. Ltd., China) following the manufacturer’s recommendations. Serum HBV-DNA was measured using a commercially available real-time fluorescent quantification PCR (FQ-PCR) diagnostic kit (Da-An Gene Co., Guangzhou, China) according to the manufacturer’s instruction.

### Immunofluorescence and western blot analysis for HBV core protein

Tissues were fixed in 4 % paraformaldehyde for 1 h, then in 30 % sucrose solution overnight for cryoprotection. 10–12 μm thick sections were cut using a Leica cryostat. Immunohistological detection of HBcAg was performed on frozen sections using primary rabbit polyclonal anti-HBcAg antibody (1:200; Abcam, England) and Alexa Fluor 488-conjugated goat anti-rabbit IgG (1:200; Jackson ImmunoResearch, USA). Finally, the sections were stained with 4′,6-diamidino-2-phenylindole (DAPI) for nuclear indication. Immunofluorescence analyses were performed with a fluorescence microscope (Leica, German).

HBV core protein was also analyzed by a standard western blot procedure using primary polyclonal anti-HBcAg antibody (1:200; Abcam, England), anti-actin monoclonal antibody (1:1000; Santa Cruz Biotechnology, USA) and peroxidase-conjugated secondary antibodies. The image was digitized using a scanner and signal was quantified using of Quantity One software (Bio-Rad).

### Statistical analysis

Each measurement was performed at least in triplicate. All data were expressed as the mean ± standard error of the mean (SEM). All statistical analyses were performed by SPSS (version 13.0 for Windows) statistical software. Differences between mean values were analyzed using One-Way Analysis of Variance (ANOVA). Statistical significance was defined by a *P* value of less than 0.05.

## Results

### Construction of the liver-targeting fusion interferon gene using *E. coli* preferred codon

**Fig. 2:**
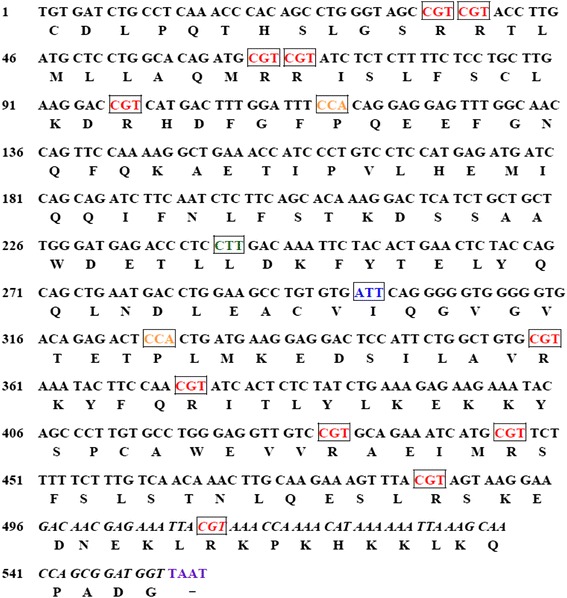
The IFN-CSP gene with codon optimization. The codons in frame are optimized

**Fig. 3:**
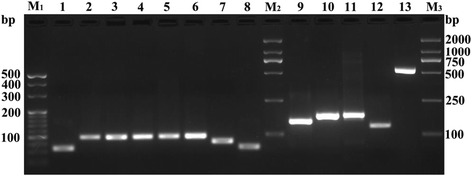
Construction of fusion gene IFN-CSP. Lane M: DNA molecular weight marker, Lane 1–8: The first PCR products ranging 68–97 bp in size, Lane 9–12: The second PCR products ranging 132–175 bp in size, Lane 13: Target amplification the full-length fusion gene IFN-CSP

To create a modified IFN-CSP gene with enhanced translation efficiency compared to the native gene, we designed a codon-optimized version of the IFN-CSP gene (GenBank Accession No. KP027473), which the rare codons of *E. coli* were changed to the preferential codons (Fig. 2). The new modified IFN-CSP gene has the identical amino acid sequence as the native gene. To increase the translation termination efficiency, the preferred stop codon TAAT was used. Finally, restriction enzyme sites for *Nde* I and *Xho* I were introduced at the 5′- and 3′-ends of the IFN-CSP gene to provide convenient restriction sites for cloning into the bacterial expression vector. Using SOE-PCR method, the synthetic IFN-CSP gene with the optimized codons was obtained (Fig. 3) and identified by DNA sequencing.

### Plasmids construction and protein expression

The above synthetic IFN-CSP gene fragment was cloned into the bacterial expression vector pET-21b to construct the expression plasmid IFN-CSP/pET-21b (Fig. 1a). The correct synthetic sequence in the recombinant plasmid IFN-CSP/pET-21b was identified by restriction enzymes with *Nde* I/*Xho* I, showing the expected about 600 bp DNA fragment (data not shown). The plasmid was then transformed into the bacterial expression host *E.coli* BL21 (DE3) to obtain recombinant strain. The target protein, which about 21.5 kD, was successfully expressed (Fig. 1b) in the form of inclusion bodies (IB).

### The effects of different expression conditions

Several one-factor experiments and an orthogonal test (L(9)(3)(4)) were employed to optimize the induction conditions.

### Cultivation temperature

**Fig. 4:**
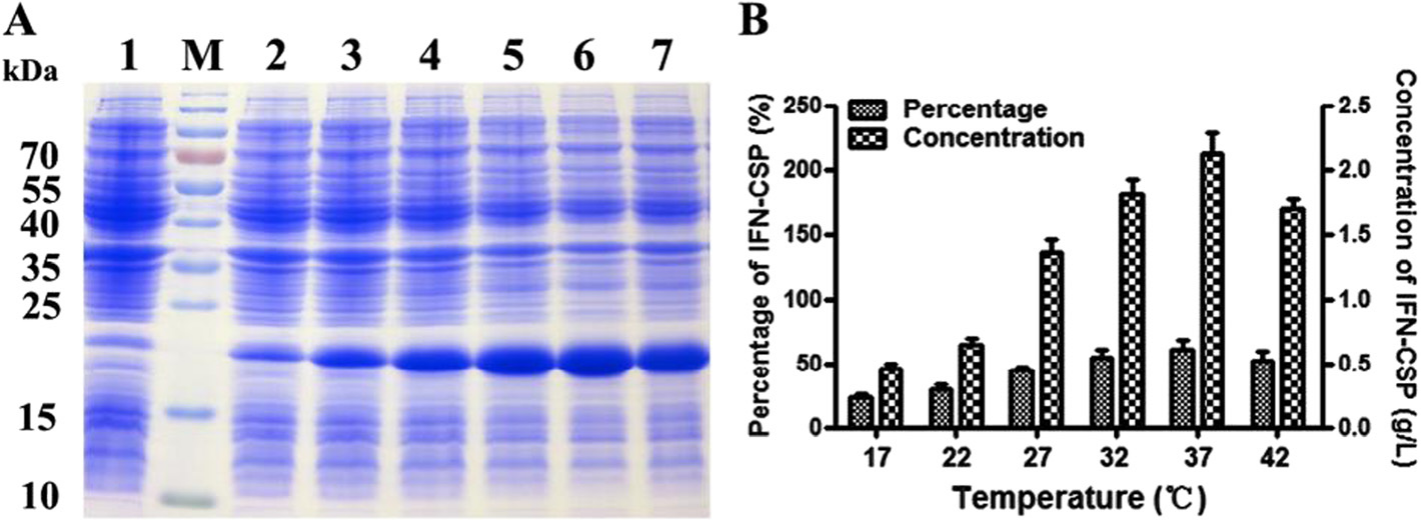
Effect of temperature on the expression level of IFN-CSP in *E. coli* BL21/pET21b-IFN-CSP. **a**: SDS-PAGE analyses of IFN-CSP expression under different temperatures. Lane M: Protein molecular weight marker; Lane 1: *E. coli* BL21/pET21b-IFN-CSP before induction; Lanes 2–7: *E. coli* BL21/pET21b-IFN-CSP was cultured at 17, 22, 27, 32, 37 and 42 °C, respectively. **b**: Percentage and concentration of IFN-CSP were calculated by the target bands in SDS-PAGE (A)

It is well-known that temperature has a great impact on the recombinant protein expression. Effects of temperature on IFN-CSP expression were investigated at 17, 22, 27, 32, 37 and 42 °C, respectively. The results (Fig. 4) revealed that IFN-CSP achieved a high percentage in total proteins and a high concentration in the temperature range of 32–37 °C.

### Induction timing

**Fig. 5:**
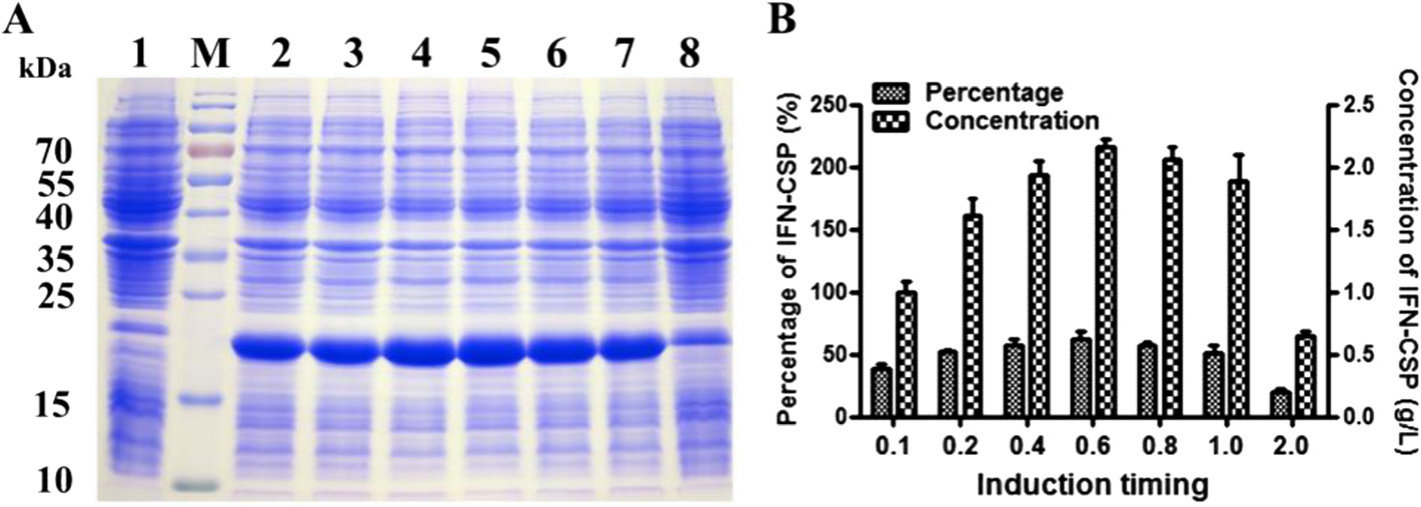
Effect of cell density on the expression level of IFN-CSP in *E. coli* BL21/pET21b-IFN-CSP. **a**: SDS-PAGE analyses of IFN-CSP expression at different growth stages. Lane M: Protein molecular weight marker; Lane 1: *E. coli* BL21/pET21b-IFN-CSP before induction; Lanes 2–8: *E. coli* BL21/pET21b-IFN-CSP was induced at OD_600_ value of 0.1, 0.2, 0.4, 0.6, 0.8, 1.0 and 2.0, respectively. **b**: Percentage and concentration of IFN-CSP were calculated by the target bands in SDS-PAGE (A)

IPTG induction initiates the translation of heterologous protein. The effect of induction timing was evaluated by adding IPTG at different stages of growth phase. The results (Fig. 5) revealed that the percentage of target protein to total proteins varied from 20.12 to 61.62% at different induction timing. The maximum percentage was observed when induced at the midexponential phase, corresponding to the OD_600_ value of 0.4–0.8.

### IPTG concentration

**Fig. 6:**
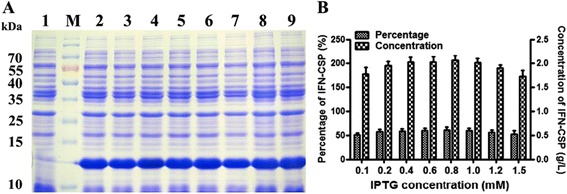
Effect of different IPTG concentration on the expression level of IFN-CSP in *E. coli* BL21/pET21b-IFN-CSP. **a**: SDS-PAGE analyses of IFN-CSP expression using different IPTG concentration. Lane M: Protein molecular weight marker; Lane 1: *E. coli* BL21/pET21b-IFN-CSP before induction; Lanes 2–9: IPTG concentrations are 0.1, 0.2, 0.4, 0.6, 0.8, 1.0, 1.2 and 1.5 mmol/L, respectively. **b**: Percentage and concentration of IFN-CSP were calculated by the target bands in SDS-PAGE (A)

Effects of final IPTG concentration on IFN-CSP expression were investigated at 0.1, 0.2, 0.4, 0.6, 0.8, 1.0, 1.2 and 1.5 mM, respectively. The results (Fig. 6) revealed that the percentage of target protein in the total proteins and the concentration of IFN-CSP achieved the highest level in the IPTG range of 0.4–0.8 mM.

### Post-induction time

**Fig. 7:**
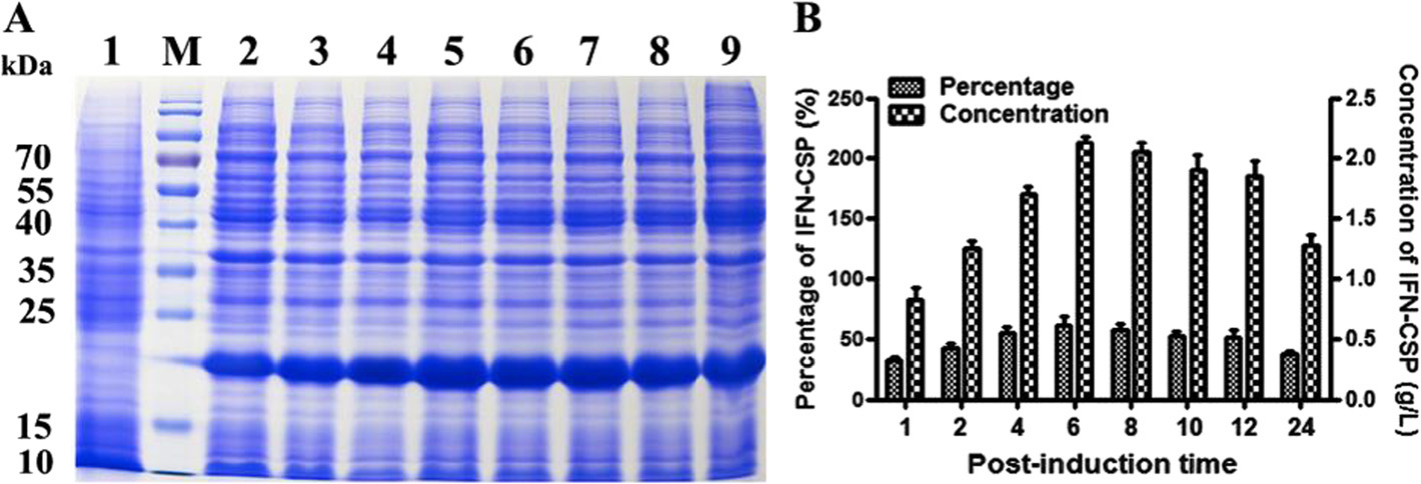
Effect of post-induction time on the expression level of IFN-CSP in *E. coli* BL21/pET21b-IFN-CSP. **a**: SDS-PAGE analyses of IFN-CSP expression at different post-induction time. Lane M: Protein molecular weight marker; Lane 1: *E. coli* BL21/pET21b-IFN-CSP before induction; Lanes 2–9: *E. coli* BL21/pET21b-IFN-CSP after induced 1, 2, 4, 6, 8, 10, 12 and 24 h, respectively. **b**: Percentage and concentration of IFN-CSP were calculated by the target bands in SDS-PAGE (A)

The results of optimal post-induction time (Fig. 7) revealed that the percentage of IFN-CSP to total proteins varied from 32.55 % to 60.89 % at different post-induction time. The maximum percentage was observed the product reached the highest concentration after a 6–10 h induction.

### Orthogonal test

**Fig. 8:**
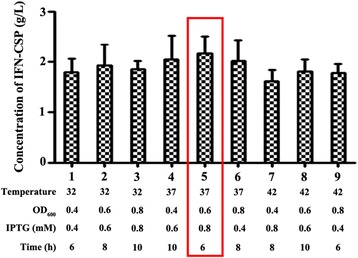
Optimization of IFN-CSP expression by orthogonal test

Based on the results of one-factor experiments, four parameters were further optimized by an orthogonal test (L(9)(3)(4)). The result of orthogonal test was showed that the optimal condition of maximum expression quantity was inducing the culture at OD_600_ = 0.6 with 0.8 mM IPTG for 6 h at 37 °C. The target protein was up to 2.17 g per L culture (Fig. 8). Main influence factor was induction temperature (data not shown).

### Purification, antigenicity and biological activity analysis

**Fig. 9:**
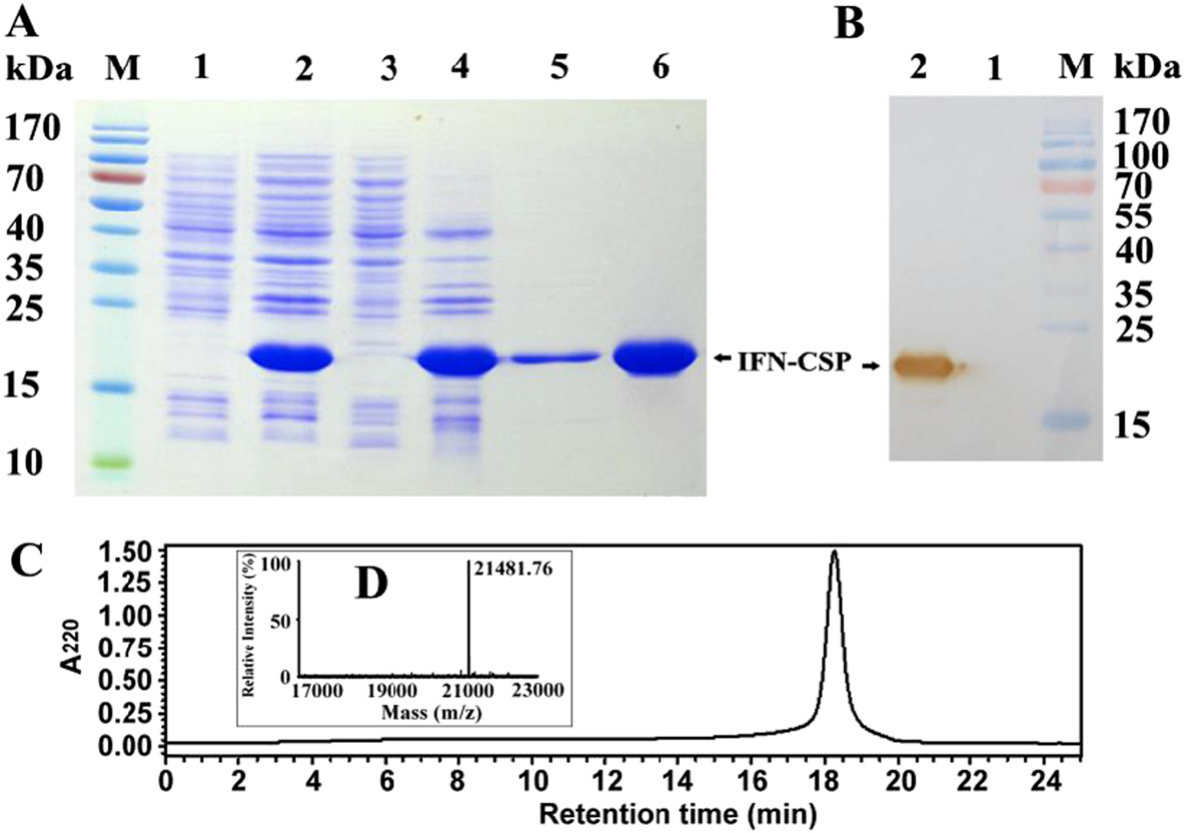
Characterization of recombinant protein by SDS-PAGE, western blot, RP-HPLC and MALDI-MS. **a**: Purification of IFN-CSP. Lane M: Protein molecular weight marker, Lane 1–2: Total proteins of *E. coli* BL21/pET-21b-IFN-CSP before and after induction, Lane 3–4: Supernatant and precipitation after ultrasonication and centrifugation. Lane 5: Purified IFN-CSP using trion and urea wash. Lane 6: Purified IFN-CSP using HiTrap affinity chromatography. **b**: IFN-CSP was analyzed by western blot. Lane M: Protein molecular weight marker. Lane 1–2: Total proteins of *E. coli* BL21/pET-21b-IFN-CSP before and after induction. **c**: Analysis of purified IFN-CSP by RP-HPLC with a C18 column. **d**: Mass spectrum of purified IFN-CSP recorded on an Applied Biosytems Voyager MALDI-TOF mass spectrometry

The total improved isolation scheme was showed in Fig. 9. The results of SDS-PAGE and RP-HPLC revealed that the target protein was purified to over 95 % homogeneity with no degradation (Fig. 9a and c). The molecular weight 21,481.76 Da determined by MALDI-MS (Fig. 9d) was consistent with its calculated value of 21,481.7 Da. Step yields were calculated and showed in Table 2. The improved isolation scheme were highly efficient in producing pure IFN-CSP, approximately 690 mg of the pure recombinant IFN-CSP was obtained from 1 L of *E. coli* culture.

**Table 2:** Isolation of IFN-CSP from E. coli inclusion bodies with a recovery protocol

Process step	Total protein	Purity	IFN-CSP	Recovery yield
	(mg/L)	(%)	(mg/L)	(%)
Cell lysis	3512	61.62	2164.09	100.00
Centrifugation	2536	79.09	2005.72	92.68
Triton wash + Urea wash	1894	85.97	1628.27	81.18
Solubilization	1690	86.39	1459.99	89.60
Refolding	1359	87.16	1184.50	81.13
HiTrap affinity	735	99.85	733.89	61.96
Dialysis	691	99.85	690.00	93.73

Total protein concentration was determined by Bradford method. Purity of IFN-CSP was calculated from densitometry analysis of SDS-PAGE gels. The concentration of IFN-CSP was calculated according the percentage fraction and total protein concentration

The results of western blot analysis revealed that purified IFN-CSP was strongly and specifically reacted with the IFN α antibody (Fig. 9b). The results of chromogenic limulus amoebocyte lysate assay showed that the LPS content in the purified sample was less than 0.5 Eu/mg proteins. Biological activity analysis using the WISH/VSV system showed that the above purified sample had ability to inhibit the cytopathic effect caused by VSV on WISH cells (3.78 × 10^8^ U/mg).

### *In vivo* tissue distribution

**Fig. 10:**
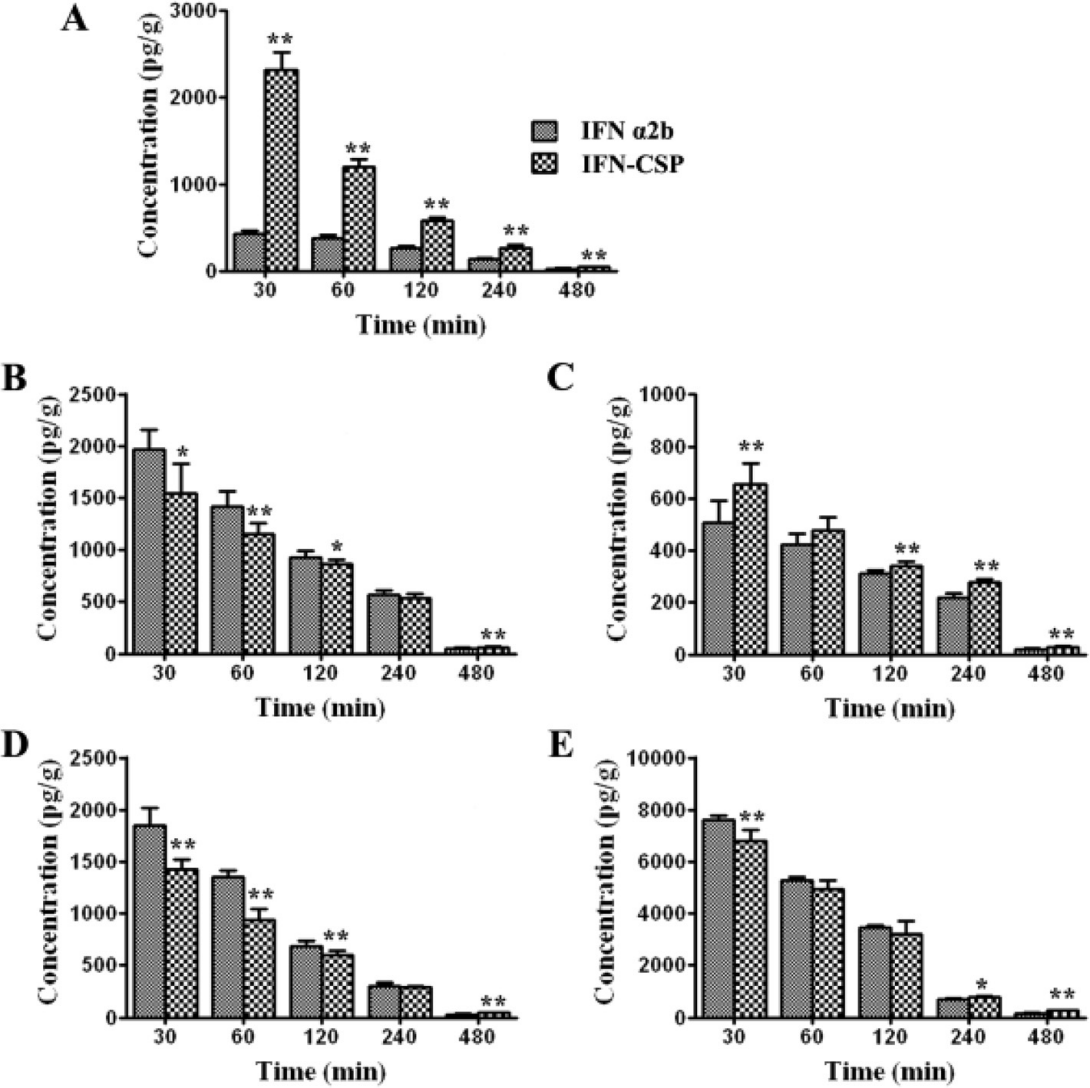
Distribution of IFN α2b and IFN-CSP in liver (**a**), heart (**b**), spleen (**c**), lung (**d**), kidney (**e**) after a single *i.v.* administration of free IFN α2b or IFN-CSP in mice at 30, 60, 120, 240 and 480 min post-injection. Each point represents the mean ± SEM (n = 6). The ** and * indicate statistically significant differences of *p* <0.01 and *p* <0.05, respectively between IFN α2b and IFN-CSP treatments

To determine whether IFN-CSP was able to accumulate in liver for potential treatment of hepatitis B, the tissue distribution of IFN-CSP were evaluated and compared to native IFN α2b. The ELISA method for IFN α2b detection is feasible and accurate (data not shown). The IFN-CSP increased the liver IFN α2b levels more significantly, up to 5.48- to 3.22-fold higher than those of native IFN α2b (*p* < 0.01) at 30, and 60 min interval post-administration (Fig. 10).

### IFN-CSP reduces serum HBsAg and HBV-DNA in HBV-transgenic mice

**Fig. 11:**
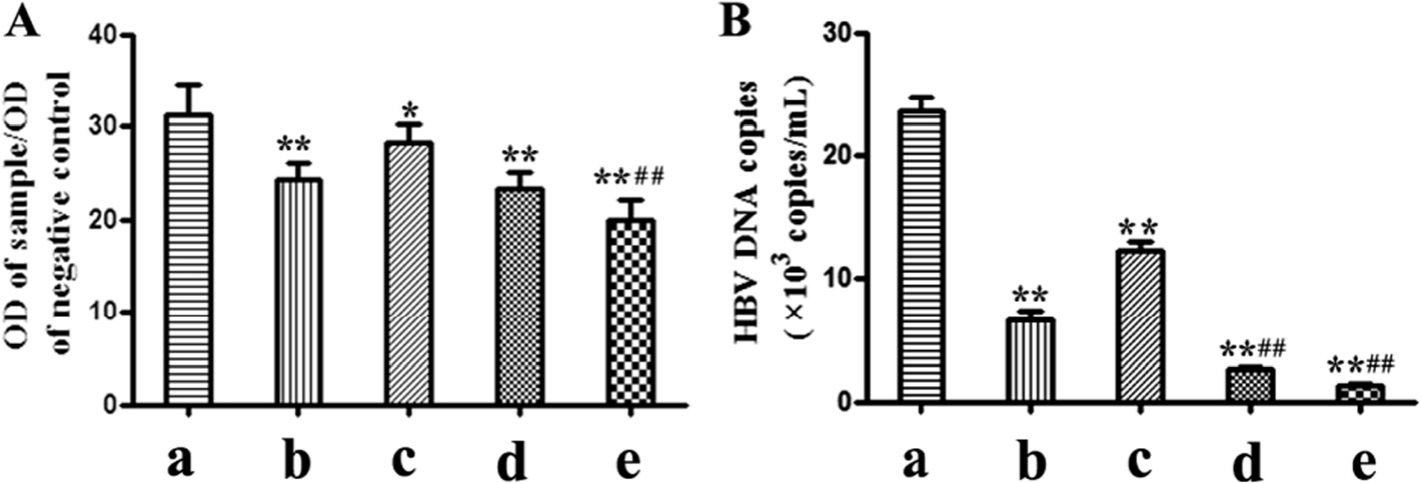
Effect of IFN α2b, IFN-CSP on serum HBsAg (**a**) and HBV-DNA (**b**) level in HBV-transgenic mice. (***a***) physiological saline control; (***b***) IFN α2b, 10^3^ U/g body weight; (***c***) IFN-CSP, 10^1^ U/g body weight; (***d***) IFN-CSP, 10^2^ U/g body weight; (***e***) IFN-CSP, 10^3^ U/g body weight. The treatment continued for 28 days, serum HBsAg levels were measured by ELISA assay. The results are represented as OD value of sample/OD value of negative control. Serum HBV-DNA levels were measured by fluorescent quantification polymerase chain reaction (PCR). The copies of the HBV-DNA were calculated based on their Ct value and the standard curves. The data are the mean ± SEM from six mice. *P < 0.05 *vs* physiological saline controls, **P < 0.01 *vs* physiological saline controls, ^# #^ *P* < 0.01 *vs* IFN α2b controls

To evaluate the *in vivo* anti-HBV activity of the recombinant IFN-CSP, Balb/c-HBV transgenic mice were treated with different concentrations of IFN-CSP for 28 days. As shown in Fig. 11, IFN-CSP reduces serum HBsAg and HBV-DNA in HBV-transgenic mice in a dose-dependent manner. Compared with the native IFN α2b group, the administration of IFN-CSP at comparable concentrations significantly reduced serum HBsAg and HBV-DNA.

### IFN-CSP reduces HBV core protein in the liver of HBV-transgenic mice

**Fig. 12:**
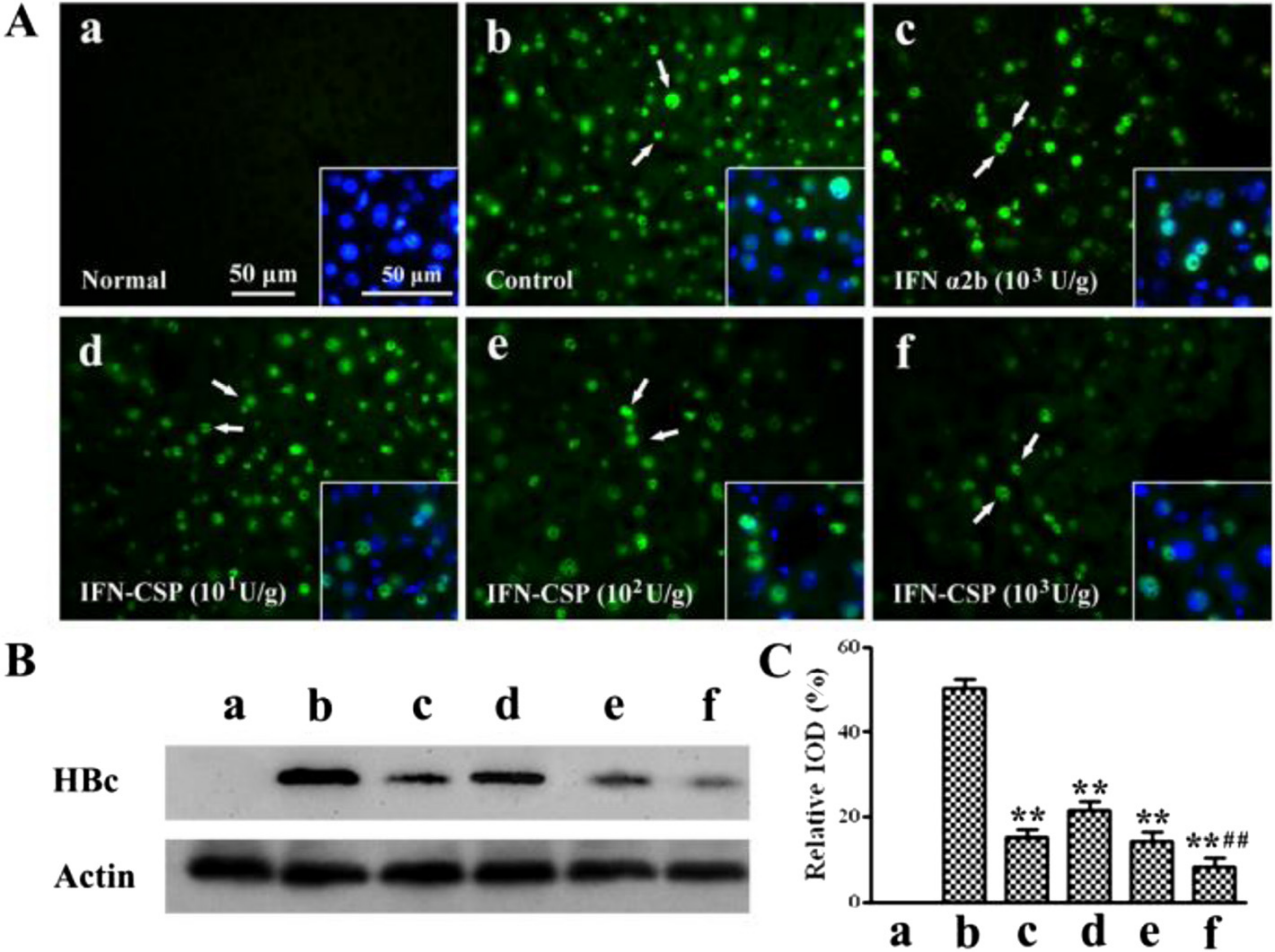
Effect of IFN α2b, IFN-CSP on HBV core protein in the liver of HBV-transgenic mice. ***a*** normal non-transgenic mice control; (***b***) physiological saline control; (***c***) IFN α2b, 10^3^ U/g body weight; (***d***) IFN-CSP, 10^1^ U/g body weight; (***e***) IFN-CSP, 10^2^ U/g body weight; (***f***) IFN-CSP, 10^3^ U/g body weight. The treatment continued for 28 days, liver of mice were collected for analyses. **a**: Representative photographs of liver sections immunofluorescent stained with anti-HBcAg antibody are presented. Arrows indicate distinct green fluorescent labeling of HBcAg in liver, blue nuclear stained with DAPI. Calibration bar =50 μm for photomicrographs. **b**: The core protein was also quantified by western blot analysis. **c**: Optical densities of the core protein were analyzed using Quantity One software. The data are the mean ± SEM from six mice. *P < 0.05 *vs* physiological saline controls, **P < 0.01 *vs* physiological saline controls, ^# #^ *P* < 0.01 *vs* IFN α2b controls

Livers from HBV-transgenic mice were detected with anti-HBcAg antibody by immunofluorescent staining and western blot analysis. The results (Fig. 12) showed that the signal of HBV core protein is obviously decreased in the liver of HBV-transgenic mice treated with IFN-CSP in a dose-dependent manner. IFN α2b control also suppressed the expression of HBV core protein, but the degree of suppression less than the same dose of IFN-CSP.

## Discussion

The expression of heterologous proteins in *E. coli* is an attractive strategy to obtain the active form of protein in a large scale for therapeutic application. In our previous work, the strategy proved to be efficient to achieve high-level expression [15, 17]. While, DNA sequence analysis of genes encoding either high abundance or low abundance proteins in *E. coli* has revealed a pattern of favored codon usage [18]. Lowly expressed genes show the greatest degree of conformity to the rare codons, which correspond to the rare tRNAs in the *E. coli* cell. So, it is necessary to analyze the DNA sequence of IFN-CSP before recombination expression using *E. coli* expression system.

The IFN-CSP contains 184 amino acids, and 15 amino acids are encoded by the rare codons of *E. coli*. Particularly, R12, R13, R22, R23, R33, R120, R125, R144, R149, and R162 were encoded by the least used codons, AGG and AGA, which were discovered to dramatically reduce the maximum level of protein synthesis. Some examples show that codon optimization can significantly improve the expression level [5, 19]. Srivastava et al. reported that a codon optimized IFN α2b gene expressed as IB only about 40 % of the total protein [20]. Valente et al. using an artificial IFN α2b gene that was codon optimized for its rare tandem arginine codons and its gene product was produced 16.6 mg/L *E. coli* culture [21]. Another investigation used *E. coli* codon optimized artificial gene produced His-IFN α2b in the form of IB -210 mg/L *E. coli* culture [22]. Based on the above knowledge, we applied a codon optimization strategy to over-express IFN-CSP fusion protein in *E. coli*. Moreover, stop codon bias and context also correlates with the efficiency of translation [23], so the preferred sequence of translation termination in *E. coli*, ‘TAAT’ was used.

**Fig. 13:**
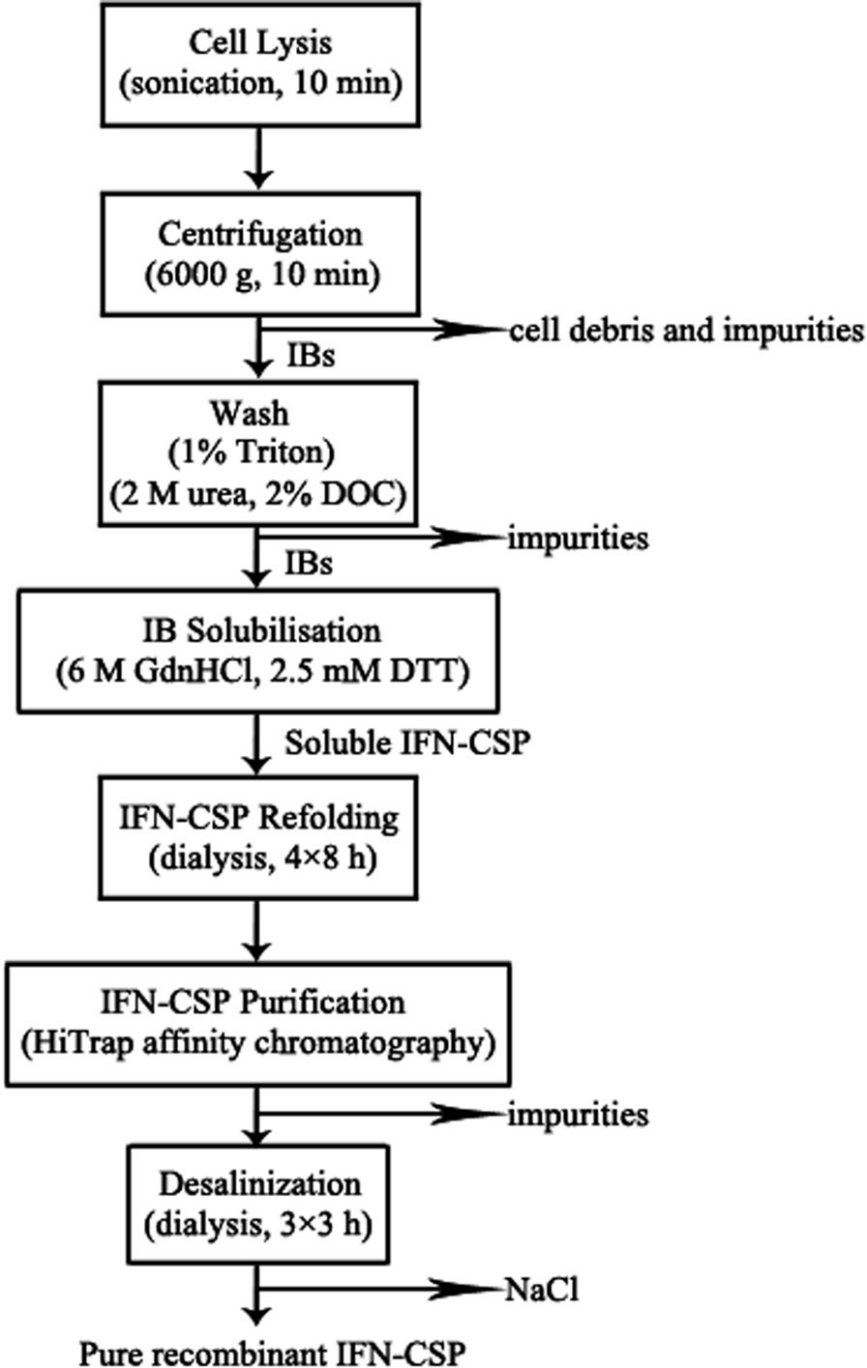
Protocol for the recovery of IFN-CSP from *E. coli* inclusion bodies

The induction conditions will affect the expression level of target proteins as reported numerous times elsewhere [16, 24]. For this reason, the induction conditions like cultivation temperature, induction timing, inducer concentrations, and induction time were also examined. In our previous study, we found that the fusion IFN-CSP without any optimization expressed as IB only accounting for 39.2 % of the total insoluble protein [13]. In the present, the highest expression level was achieved when the recombinant *E. coli* at the mid-exponential phase was effectively induced at 37 °C with 0.8 mM IPTG concentration for 6 h. Although most of the IFN-CSP protein in the current work was produced in the form of IB (Fig. 1b), this is the first report to show that the novel liver-targeting fusion interferon (IFN-CSP) was expressed in such high level, about 61.62 % relative to total host proteins. Using the improved purification scheme (Fig. 13), the final yield of purified IFN-CSP was up to 690 mg per L culture, which is 2.56-fold higher than the previous investigation [13]. The purity and the molecular weight of purified IFN-CSP were demonstrated by SDS-PAGE, RP-HPLC and MALDI-MS (Fig. 9). The result of western blot analysis characterized the antigenicity of IFN-CSP (Fig. 9b). Biological activity analysis according to China Biologicals Requirements showed that IFN-CSP had ability to inhibit the cytopathic effect caused by VSV on WISH cells (3.78 × 10^8^ U/mg).

In line with previous *in vitro* experiments, we have further evaluated the *in vivo* tissue distribution and anti-HBV activities of IFN-CSP. *In vivo* tissue distribution assay showed that IFN-CSP was able to accumulate in mouse liver compared to native IFN α2b (Fig. 10). Small animal models amenable to infection with human hepatotropic viruses are in great need for studying new anti-HBV drugs. The HBV-Tg mouse lineage was initially produced on a BALB/c background. The transgene in these mice consists of 1.3 copies of the HBV ayw complete genome. The HBV-Tg mice express high level of HBsAg in their serum and have detectable HBV-DNA in their serum, which allows HBV-DNA replication, is an appropriate tool to evaluate anti-HBV drugs [25–28]. In the present study, we found that the recombinant IFN-CSP and native IFN α2b *in vivo* were able to reduce serum HBsAg (Fig. 11a), HBV-DNA (Fig. 11b) and liver HBV core protein (Fig. 12) in HBV-transgenic mice. However, IFN-CSP showed a significant improvement in anti-HBV activity when compared with native IFN α2b at similar doses. These results indicate that incorporation of *plasmodium* region I peptide can specifically target to liver, to achieve higher local concentrations and to improve their anti-HBV efficacy *in vivo*.

## Conclusion

In this report, synthetic codon optimized IFN-CSP gene was overexpressed at high expression level and the purified protein was confirmed to IFN-CSP based on the results of antigenicity and biological activity analysis. The purified IFN-CSP was able to accumulate in mouse liver compared to native IFN α2b and reduce serum HBsAg, HBV-DNA and liver HBV core protein in HBV-transgenic mice. This current work reports for the first time that codon and expression conditions optimized approach is a strategy to produce IFN-CSP at large scale, and would also be very helpful to produce other recombinant protein for further medical research. The present study further supported the application of IFN-CSP in liver-targeting anti-HBV medicines.

## Abbreviations

IFN-CSP: Liver-targeting fusion interferon (fusing interferon with circumsporozoite protein region I-plus)
HBV: Hepatitis B virus
*E. coli*: *Escherichia coli*
IPTG: Isopropyl β-D-1-thiogalactopyranoside
IFNs: Interferons
PCR: Polymerase chain reaction
SOE-PCR: Splicing by overlapping extension-PCR
LB: Luria-Bertani
6-His: 6-histidine
SDS-PAGE: Sodium dodecyl sulphate polyacrylamide gel electrophoresis
GuHCl: Guanidine hydrochloride
LPS: Lipopolysaccharides
VSV: Vesicular stomatitis virus
WISH: Human amniotic cells
HBsAg: Hepatitis B s antigen
HBcAg: Hepatitis B core antigen
ELISA: Enzyme-linked immunosorbent assay
FQ-PCR: Fluorescent quantification PCR
DAPI: 4′,6-diamidino-2-phenylindole
SEM: Standard error of the mean
ANOVA: Analysis of Variance
IB: Inclusion bodies
